# Cardiovascular Disease-Related Parameters and Oxidative Stress in SHROB Rats, a Model for Metabolic Syndrome

**DOI:** 10.1371/journal.pone.0104637

**Published:** 2014-08-12

**Authors:** Eunice Molinar-Toribio, Jara Pérez-Jiménez, Sara Ramos-Romero, Laura Lluís, Vanessa Sánchez-Martos, Núria Taltavull, Marta Romeu, Manuel Pazos, Lucía Méndez, Aníbal Miranda, Marta Cascante, Isabel Medina, Josep Lluís Torres

**Affiliations:** 1 Institute of Advanced Chemistry of Catalonia (IQAC-CSIC), Barcelona, Spain; 2 Biomedical Research Networking Center in Bioengineering, Biomaterials, and Nanomedicine (CIBER-BBN), Zaragoza, Spain; 3 Unidad de Farmacología, Facultad de Medicina y Ciencias de la Salud, Universidad Rovira i Virgili, Reus, Spain; 4 Instituto de Investigaciones Marinas (IIM-CSIC), Vigo, Spain; 5 Department of Biochemistry and Molecular Biology, IBUB and unit associated with CSIC, Faculty of Biology, Universitat de Barcelona, Barcelona, Spain; 6 Institut d'Investigacions Biomèdiques August Pi i Sunyer (IDIBAPS), Barcelona, Spain; University of Southampton, United Kingdom

## Abstract

SHROB rats have been suggested as a model for metabolic syndrome (MetS) as a situation prior to the onset of CVD or type-2 diabetes, but information on descriptive biochemical parameters for this model is limited. Here, we extensively evaluate parameters related to CVD and oxidative stress (OS) in SHROB rats. SHROB rats were monitored for 15 weeks and compared to a control group of Wistar rats. Body weight was recorded weekly. At the end of the study, parameters related to CVD and OS were evaluated in plasma, urine and different organs. SHROB rats presented statistically significant differences from Wistar rats in CVD risk factors: total cholesterol, LDL-cholesterol, triglycerides, apoA1, apoB100, abdominal fat, insulin, blood pressure, C-reactive protein, ICAM-1 and PAI-1. In adipose tissue, liver and brain, the endogenous antioxidant systems were activated, yet there was no significant oxidative damage to lipids (MDA) or proteins (carbonylation). We conclude that SHROB rats present significant alterations in parameters related to inflammation, endothelial dysfunction, thrombotic activity, insulin resistance and OS measured in plasma as well as enhanced redox defence systems in vital organs that will be useful as markers of MetS and CVD for nutrition interventions.

## Introduction

The combination of CVD risk factors known as metabolic syndrome (MetS) is becoming a major public health problem which now affects 20%–30% of the adult population in developed countries [1]. The risk factors include abdominal obesity, hyperglycaemia, hypertension and/or hypertriglicerydaemia associated with a sedentary lifestyle [2]. The main underlying disorder in MetS appears to be insulin resistance, i.e., an impairment of insulin action within tissues (mainly muscle, liver and adipose tissues) that has been related to chronic low-level inflammation as well as oxidative stress (OS), as second-order underlying mechanisms for this pathological condition [3], [4]. The factors defining MetS may lead to the onset of type-2 diabetes and CVD. This is the main reason why the study of the factors underlying MetS can be clinically useful.

There is wide interest in the development of strategies for the prevention of MetS, mainly via two different approaches: a) pharmacology – based on the use of drugs such as certain statins or AMPK (5′ adenosine monophosphate-activated protein kinase) activating agents [5], [6]; and b) nutrition–based on diet supplementation with functional compounds, e.g., proanthocyanidins [7] or iminosugars [8].

To test the efficacy of the different approaches, several animal models of MetS have been suggested, in which MetS is either induced by the diet [9], [10] or results from some genetic alteration. Most of the genetically modified animals present mutations in the gene that codes for the leptin receptor [11], which impairs the capacity of leptin to regulate food intake and eventually leads to the development of insulin resistance. One such model is SHROB (spontaneously hypertensive obese) rats, also called Koletsky rats [12], [13]. SHROB rats were generated by crossing a female spontaneously hypertensive rat (SHR), obtained by breeding high-blood-pressure Wistar-Kyoto rats, with a normotensive Sprague Dawley male, from which a single homozygous recessive trait appeared. That trait is a nonsense mutation in the leptin receptor, designated fa^k^, which results in a premature stop codon in the extracellular domain of the leptin receptor that circumvents the process of transduction and thereby truncates all the leptin receptors [14], [15]. SHROB rats, homozygous for this mutation, are therefore characterized by monogenetic obesity combined with a hypertensive background for which several phenotype aspects have been described, including hyperinsulinaemia, hyperlipidaemia and nephropathy [15]. SHROB rats are thus a model for MetS, in accordance with some of its currently accepted defining factors, i.e., insulin resistance, hypertension, increased plasma triglyceride levels and obesity [16]. It has also been suggested that in SHROB rats hyperinsulinaemia is not accompanied by hyperglycaemia, this model would be especially useful for the study of the prediabetic state [17], in contrast to other animal models (e.g. Zucker rats) which develop type-2 diabetes.

Despite its use as a model of MetS, a complete description of the SHROB rat phenotype, in relation to markers related to CVD and particularly OS, is lacking. Moreover, studies with SHROB rats have compared them with SHR, spontaneously hypertensive rats [15], [18], a rat that is already mutated and in which several parameters are altered, and not with either of the original rat strains from which these rats were derived, i.e., Sprague-Dawley or Wistar Kyoto (WKY) rats.

The aim of this work is to complete a phenotypic description of SHROB rats using previously unexplored parameters related to CVD, and compare it with that of non-mutated WKY rats. We obtained new data of relevance for the use of SHROB rats in nutrition studies.

## Materials and Methods

### Materials and reagents

The A04 diet was from Harlan Iberica (Panlab S.L., Barcelona, Spain). Soybean oil, obtained from unrefined organic soy oil (first cold pressing), was from Clearspring Ltd. (London, UK).

Ketamine chlorhydrate was from Merial Laboratorios (Barcelona, Spain). Xylacine was from Quimica Farmaceutica (Barcelona, Spain). [2H4]-15-F2t-IsoP, 15-F_2t_-IsoP and 15-F_2t_-IsoP methyl ester were obtained from Cayman Chemical (Ann Arbor, MI, USA). 2,3,4,5,6-Pentafluorobenzyl (PFB) bromide, N,N-diisopropylethylamine (DIPE), acetonitrile, anhydrous sodium sulphate, heptane, phenylmethylsulphonyl fluoride (PMSF), dithiothreitol (DTT), iodoacetamide, ethylenediaminetetraacetic acid (EDTA), Tris-HCl, 3,3-cholaminopropyl-dimethylammonio-1-propanesulphonate (CHAPS), anhydrous magnesium chloride, bicinchoninic acid (BCA), 2,2′-Azobis(2-amidinopropane) dihydrochloride (AAPH), 6-hydroxy-2,5,7,8-tetramethylchroman-2-carboxylic acid (Trolox), fluorescein (sodium salt), acetone, phosphomolybdic acid, Bradford reagent, NADPH (β- Nicotinamide adenine dinucleotide 2′-phosphate reduced tetrasodium salt hydrate), glutathione reductase (GR from baker's yeast), L-glutathione reduced (GSH minimum 99%), t- tert-butyl hydroperoxide (t-BuOOH)) and L-glutathione oxidized (GSSG) were all obtained from Sigma-Aldrich (St. Louis, MO, USA). N,O-bis(trimethylsilyl)trifluoro-acetamide (BSTFA) was from Supelco (Bellefonte, PA, USA). Hydrochloric acid fuming 37%, ethanol absolute, ethyl acetate, methanol, chloroform, Na_2_CO_3_, NaHCO_3_, EDTA Na^2+^, o-phthalaldehyde (OPT), N-ethylmaleimide (NEM) and thiobarbituric acid were purchased from Merck KGaA (Darmstadt, Germany). Fluorescein-5-thiosemicarbazide (FTSC) was purchased from Invitrogen (Carlsbad, CA, USA). ProteoBlock protease inhibitor cocktail was purchased from Thermo Fisher Scientific Inc. (Rockford, IL, USA). Urea, thiourea, SDS, glycine and glycerol were obtained from USB (Cleveland, OH, USA). Ammonium persulphate (APS), IPG buffer, pharmalyte 3-10; bromophenol blue and 1,2-bis(dimethylamino)-ethane (TEMED) were purchased from GE Healthcare Bio-Sciences AB (Uppsala, Sweden). Acrylamide, bis (N,N′-methylene-bis-acrylamide) and the Bio-Rad protein assay were obtained from Bio-rad (Hercules, CA, USA). Drabkin Reagent and albumin were purchased from Química Clinica Aplicada (Tarragona, Spain). Na_2_HPO_4_, NaH_2_PO_4_, NaCl, epinefrine, KH_2_PO_4_, K_2_HPO_4_, trichloroacetic acid (TCA) and NaOH were purchased from Panreac Química (Barcelona, Spain). Water for the assay solutions was obtained using a Milli-Q water purification system from Millipore Corporation (Billerica, MA, USA).

### Animals

Seven female, 11- to 14-week-old, spontaneously hypertensive obese rats (SHROB) (Charles River Laboratories, Wilmington, MA, USA) and seven female, 11-week-old WKY rats (control group) (Janvier, Le Genest-St-Isle, France) were used. They were kept away from male rats to minimize hormonal alterations.

### Experimental design

The rats were kept in Macrolon cages (*n* = 2–3/cage, 425×265×180 mm) under controlled conditions of stable humidity (50±10%), and temperature (22±2°C) with a 12∶12-h light/dark cycle. All the groups were fed a standard pelleted A04 diet, given water (Ribes, Barcelona, Spain) *ad libitum* and administered a weekly oral dose of 0.8 mL/kg of soybean oil for 13-weeks-fatty acid composition provided as **Table S1**. The additional dose of fat (weekly excess of 5–10% of fat intake) was intended to accelerate the appearance of alterations in CV risk factors and OS related parameters. At week 15, one week after the last intragastric dose of soybean oil and oral glucose tolerance test (OGTT)—see below—the rats were fasted overnight, anesthetized intraperitoneally with ketamine and xylacine (80 mg/kg and 10 mg/kg body weight respectively), and then killed by exsanguination. All the procedures follow the European Union guidelines for the care and management of laboratory animals and all efforts were made to minimize suffering. The pertinent permission for this specific study was obtained from the CSIC (Spanish Research Council) Subcommittee of Bioethical Issues (ref. AGL2009-12 374-C03-03).

### Sample collection

For urine collection, at week 12 of the experiment, the rats were placed in metabolic cages and deprived of food for 18 h; the animals had previously been acclimated to the metabolic cages. At the end of the experimental period, after one week of recuperation since the final intragastric dose of soybean oil, blood was collected by cardiac puncture under anaesthesia. Plasma, serum and erythrocytes were collected. Tissue samples collected from heart, brain, liver, and kidney were washed with 0.9% NaCl solution and immediately frozen in liquid nitrogen. Abdominal fat was weighed and immediately frozen. All the samples were stored at −80°C pending analysis. Before analysis, tissue samples were homogenized with sodium phosphate buffer and ultra-centrifuged.

### Cardiovascular disease risk factors

Body weight was monitored weekly throughout the experiment.

#### Determination of plasma lipid profile

Total plasma cholesterol (CHOL), LDL-cholesterol (LDLc), HDL-cholesterol (HDLc) and triglycerides (TG) were measured by spectrophotometric methods using the corresponding kits from Spinreact (Girona, Spain). ApoA1 and ApoB100 were measured using ELISA kits purchased from Cusabio Biotech (Hubei, China)

#### Determination of glycaemia

Blood glucose levels were measured by the enzyme electrode method using an Ascensia ELITE XL blood glucose meter from (Bayer Consumer Care (Basel, Switzerland). Plasma insulin was measured using ELISA kits from Millipore (Billerica, MA, USA). Homeostasis model assessments (HOMA) were calculated by applying the formula: fasting plasma glucose (mg/dL) times fasting insulin (mU/L) divided by 405 [19]. Glycated Hb was measured using a spectrophotometric kit from Spinreact. All these measurements were carried out at the end of the study.

An OGTT was conducted at week 14 of the experiment after 18 h of food deprivation. Rats were administered a glucose dose of 2 g/kg body weight by gastric probe using a 29% glucose solution. The glucose concentration was measured by the enzyme electrode method, using single drops of blood taken from the saphenous vein at 0, 15, 30, 45, 60, 90 and 120 min after glucose administration. The AUC for serum glucose was calculated over the 2 h period. This test was carried out 1 week after the last intragastric dose of soybean oil.

#### Blood pressure

Systolic and diastolic blood pressure were measured at week 13 of the experiment. In a quiet place, at an established time to reduce circadian rhythm interference, the rats were restrained in a rat pocket and maintained at 32°C. Systolic and diastolic blood pressure were measured three times by the tail-cuff method, using a non-invasive automatic blood pressure analyser (Panlab, Barcelona, Spain) as previously described [20]. Data are presented as the mean of three measurements.

#### Markers of endothelial function, inflammation and thrombotic activity

The corresponding ELISA kits from Cusabio Biotech (Hubei, China) were used to measure the following parameters in plasma: vascular cell adhesion molecule-1 (VCAM-1) and intercellular adhesion molecule-1 (ICAM-1) as markers of endothelial function; C-reactive protein (CRP) as an inflammation marker.; plasminogen activator inhibitor-1 (PAI-1) as a marker of thrombotic activity.

### Endogenous antioxidant systems

#### Activity of superoxide dismutase (SOD) and catalase (CAT)

SOD and CAT activities were measured using standard spectrophotometric methods [21], [22] in the following samples: erithrocytes (0.23–1.84 µL and 0.5 µL, respectively), liver (167–417 µg and 1.25 mg, respectively), abdominal fat (1.6–4 µg and 12 mg, respectively), kidney (667–1667 µg and 10 mg, respectively), heart (333–833 µg and 5 mg, respectively) and brain (833–417 µg and 5 mg, respectively).

#### Glutathione system

Glutathione peroxidase (GPx) and glutathione reductase (GR) activities were measured by spectrophotometric methods [23], [24]. Glutathione (GSH) and glutathione disulphide (GSSG) were determined using fluorometric methods [25].

GR and GPx analysis were measured in the following samples: erythrocytes (8–48 µL ), liver (40–240 µg), abdominal fat (2–12 mg), kidney (80–480 µg), heart (80–480 µg) and brain (500–3000 µg). GSH and GSSG were measured in erythrocytes (149 µL), liver (75 µg), abdominal fat (372 µg), kidney (298 µg), heart (149 µg) and brain (372 µg). The standard curves used for the quantification of GSH and GSSG are provided as **Figures S1 and S2**.

In both cases, the activities of the enzymes were normalized to the amount of protein by the Bradford method [26] and to the amount of Hb in erythrocytes by the Drabkin method [27].

### Oxidative stress

#### Determination of oxygen radical absorbance capacity

Total plasma antioxidant capacity was measured as the oxygen radical absorbance capacity (ORAC) [28], which measures the level of protection from the loss of intensity of the fluorescent molecules fluorescein, due to the action of peroxyl radicals generated from an azo-initiator. A standard curve for Trolox (reference compound for antioxidant capacity determination) was prepared in the range 12.5–100 µM.

#### Protein carbonylation

Protein concentrations in the homogenates were determined according to the method of Bradford [26], using a standard curve between 0.125 and 0.975 mg protein/mL standard. Preparation of homogenates from liver aliquots, determination of the protein concentration in the homogenates and protein separation by 10% SDS-PAGE proteins from 250 mg liver were fractionated according to hydrophobicity, into proteins soluble at low ionic strength, mostly water-soluble proteins, and proteins soluble at high ionic strength, mostly hydrophobic proteins. After homogenization, each liver fraction (300 µL) and plasma (25 µL) were independently incubated with 1 mM fluorescein-5-thiosemicarbazide (FTSC) in the dark as previously described [29], [30]. After incubation, the proteins were precipitated with an equal volume of 20% TCA (v/v), centrifuged and finally, re-dissolved in urea buffer (7 M urea, 2 M thiourea, 2% CHAPS, 0.5% Pharmalyte 3–10, 0.5% IPG 3–10 buffer and 0.4% DTT). To evaluate the protein carbonyl levels, 30 µg of each sample were subjected to one-dimensional (1-DE) 10% SDS-PAGE [31] and run in a Mini-protean 3 cell (Bio-Rad). After electrophoresis, FTSC-tagged proteins were visualized by exposing the gel to a UV transilluminator Molecular Imager Gel Doc XR System (Bio-Rad) equipped with a 520 nm band-pass filter (520DF30 62 mm) and the scanned one-dimensional gels were analysed with the 1-D gel analysis software LabImage 1D from Kapelan Bio-Imaging Solutions (Halle, Germany). Finally, the scanned analysed gels were stained overnight with the Coomassie dye PhastGel Blue R-350 (GE Healthcare, Little Chalfont, UK) to visualize the total amount of protein in each sample. The global protein carbonylation level for each sample was quantified based on the overall optical volume of each lane in the FTSC-stained 1-D electrophoresis gel, whereas the oxidation level of a specific protein band was estimated by its optical intensity (peak height). Oxidized albumin (1 to 14 µg) was used as a positive control.

#### Oxidized LDL

Plasma oxidized LDL (LDL-ox) was determined with an ELISA kit from Cusabio Biotech, by measuring the absorbance at 450 nm on a PowerWave XS2 spectrophotometer from BioTec Instruments, Inc., (Winooski, VT, USA).

#### Malondialdehyde (MDA)

MDA concentrations were measured in homogenized liver (80 µg of sample), kidney (80 µg), abdominal fat (80 µg), heart (10 µg) and brain (400 µg) with fluorescence detection [32]. The fluorescence at 515 nm excitation wavelength and 548 nm emission wavelength was determined on an LS50B spectrofluorimeter (Perkin Elmer, Alabama, USA).

#### Determination of isoprostane 15-F_2_t-IsoP

Urine 15-F2t-IsoP was determined by GC/negative ion chemical ionization/MS (GC-NICI-MS) following the method of Milne *et al*. [33]. Briefly, after addition of the deuterated internal standard [2H4]-15-F2t-IsoP to 250 µL of urine, the samples were subjected to solid phase extraction (SPE) purification, with a C18 Sep-Pak column and a silica Sep-Pak column (both from Waters Associates, Milford, MA, USA). The F_2_-Isoprostanes were derivatized to pentafluorobenzyl (PFB) esters and further purified by thin layer chromatography (TLC) using 15-F_2t_-IsoP methyl ester as the reference compound with an *R*_F_ value identical to the analyte. Finally, the pure derivatized analytes were converted to trimethylsilyl ether derivatives and analysed by GC-NICI-MS using a Shimadzu QP201 instrument (Tokyo, Japan), fitted with a DB1701 fused silica capillary column (15 m×0.25 mm i.d., 0.25 µM film thickness) from Agilent (J & W Scientific, Folsom, CA, USA). The 15-F2t-IsoP ion monitored was the carboxylate anion at *m/z* 569. Representative chromatograms used for the determination of 15-F2t-IsoP are provided as **Figure S3**. The final results are expressed in ng/mg creatinine. Creatinine levels from the urine samples were determined by a colorimetric method using a commercial kit from C-cromatest Linear Chemicals (Barcelona, Spain) by measuring absorbance at 510 nm on a SpectraMax M5 spectrophotometer (Molecular Devices, Sunnyvale, CA, USA).

### Statistical analysis

The different analytical determinations in the biological samples were carried out in duplicate or triplicate, depending on the method. Results are expressed as the mean + SEM. The non-parametric Mann-Whitney U's test was applied to analyse the significant differences (*P*<0.05) between groups. The SPSS IBM19 package for Windows was used throughout.

The weight distribution between the groups of rats at each of the 15 time points was compared using the Wilcoxon Rank Sum test. Due to the large number of comparisons, a multi-test procedure that applies multiple comparisons as is known as the false discovery rate (FDR) [34] correction, with Simes' method [35] was used. With this procedure a comparison was considered statistically significant if the corresponding p-value was lower than or equal to the specific α-corrected value for the comparison. This analysis was performed using the statistical software STATA 12.0.

## Results

### Cardiovascular disease risk factors

The SHROB rats were already obese at week 16, with a body weight twice that of the WKY rats (483±9 g versus 221±4 g). Moreover, while the WKY rats maintained a constant body weight up to week 27, the SHROB rats showed an increase from 476.8±15.9 g at week 16 to 605.6±19.5 g at week 27. **Figure S4** shows the evolution of body weight of the SHROB and WKY rats during the study.

Abdominal fat at the end of the study, expressed as percentage of body weight was 50% higher in the SHROB than in the WKY rats (**Table 1**).

**Table 1:** CVD risk factors evaluated in the plasma of SHROB and WKY rats (mean value and SEM)^1^.

Category	Parameter	SHROB		WKY	
			SEM		SEM
Physical measurements	Abdominal fat (% bw)	6·7^**^	0·4	4·3	0·2
Plasma lipid profile	Total cholesterol (mM)	8·3^**^	1·2	3·3	0·2
	LDL-cholesterol (mM)	0·4^*^	0·1	0·1	0·004
	HDL-cholesterol (mM)	2·9^*^	0·2	1·9	0·1
	Total triglycerides (mM)	40·0^**^	0·0	5·6	1·1
	Apo A1 (mg/dL)	68·3^*^	1·9	51·4	5·0
	Apo B100 (mg/dL)	205·4^*^	18·7	126·8	5·3
Glycaemia	Glucose (mM)	3.4	0.1	3.5	0.1
	Insulin (nM)	1·0^**^	0·2·9	0·3	0·1
	HOMA index	15·6^**^	2·4	5·6	1·1
	OGTT (AUC)	5016·3	177·4	4494·0	122·8
	Glycated Hb (%)	6·8	0·4	6·4	0·4
Blood pressure	Systolic blood pressure (mmHg)	193·2^**^	12·5	121·0	2·2
	Diastolic blood pressure (mmHg)	134·8^**^	5·2	106·5	1·7
Endothelial dysfunction	VCAM-1 (µg/mL)	2·7	0·3	3·4	0·6
	ICAM-1 (ng/mL)	1·0^**^	0·1	0·5	0·03
Inflammation	C reactive protein (pg/mL)	285·9^**^	22·0	172·8	14·4
Thrombotic activity	PAI-1 (µg/mL)	11·6^**^	0·5	6·4	0·3

1 SHROB, Spontaneously Hypertensive Obese; WKY, Wistar Kyoto; OGTT, oral glucose tolerance test; VCAM-1, vascular cell adhesion molecule-1; ICAM-1, intercellular adhesion molecule-1; PAI-1, plasminogen activator inhibitor-1.

Statistically significant differences between the samples are indicated by * (*P*<0.05), ** (*P*<0.01) and *** (*P*<0.001).

#### Plasma lipid profile

The SHROB rats presented significantly higher (*P*<0.01) levels of plasma total cholesterol, LDL-cholesterol, HDL-cholesterol and triglycerides than the WKY rats (**Table 1**). In particular, the SHROB rats showed a ninefold increase in total cholesterol and more than a tenfold increase in triglycerides, compared to the WKY rats. Plasma levels of both the anti-atherogenic ApoA1 and the pro-atherogenic Apo B100 were also significantly higher (*P*<0.01) in the SHROB rats than in the WKY rats (+33% and +62%, respectively).

#### Glycaemia

No significant differences were observed in fasting plasma glucose, OGTT or in glycated Hb between the SHROB and WKY rats. In contrast, plasma insulin was significantly higher (*P*<0.01) in the SHROB rats than in the WKY rats (1.0±0.3 nM versus 0.3±0.1 nM, respectively), and consequently, the HOMA index was also higher in the SHROB rats (**Table 1**).

#### Blood pressure

Both systolic and diastolic blood pressure were significantly higher (*P*<0.001) in the SHROB rats (193±12 mmHg and 135±5 mmHg, respectively) than in the WKY rats (121±2 mmHg and 106±2 mmHg) (**Table 1**).

#### Endothelial dysfunction

ICAM-1 and VCAM-1 were measured as markers of endothelial dysfunction; considered the earliest stage in the atherosclerosis process. ICAM-1 showed an 84% increase in plasma levels in the SHROB rats compared to the WKY rats (*P*<0.001). No significant differences were found in plasma VCAM-1 between the SHROB and WKY rats (**Table 1**).

#### Inflammation

Plasma C-reactive protein, determined as a marker of inflammation, was 65% higher in the SHROB rats than in the WKY rats (*P*<0.001) (**Table 1**).

#### Thrombotic activity

The SHROB rats presented significantly higher (*P*<0.001) plasma levels of PAI-1 than the WKY rats (6.4±0.3 µg/mL versus 11.6±0.5 µg/mL, respectively) (**Table 1**).

### Endogenous antioxidant systems

Endogenous antioxidant systems were evaluated in erythrocytes, tissues and organs of the SHROB and WKY rats at the end of the study (**Table 2**).

**Table 2:** Endogenous antioxidant systems in SHROB and WKY rats (mean value and SEM)^1^.

Organ	Parameter	SHROB		WKY	
			SEM		SEM
Erythrocytes	SOD (U/g Hb)	768·0	224·0	1444·0	161·0
	CAT (mmol/min/g Hb)	51·9	4·1	41·5	13·1
	GR (U/g Hb)	0·9	0·1	0·9	0·1
	GPx (U/g Hb)	51·5^*^	10·3	101·5	14·4
Liver	SOD (U/g mg prot)	8·3	0·5	8·7	0·6
	CAT (µmol/min/mg prot)	70·8	9·8	81·1	10·9
	GR (mU/mg prot)	66·7^*^	3·1	88·8	8·3
	GPx (mU/mg prot)	245·4^*^	31·0	383·8	37·1
	GSH (nmol/mg prot)	61·2^**^	9·6	29·9	2·6
	GSSG (nmol/mg prot)	133·7	12·1	61·2	9·6
	GSH/GSSG	0·5^*^	0·1	0·5	0·1
Abdominal fat	SOD (U/g mg prot)	4·2	0·7	6·5	1·4
	CAT (µmol/min/mg prot)	27·8	4·9	28·3	2·5
	GR (mU/mg prot)	31·2	7·3	28·3	2·1
	GPx (mU/mg prot)	38·7^**^	2·6	15·8	4·0
	GSH (nmol/mg prot)	110·4^*^	24·9	41·7	6·4
	GSSG (nmol/mg prot)	49·8	4·8	47·7	3·0
	GSH/GSSG	2·2^*^	0·5	0·9	0·1
Kidney	SOD (U/g mg prot)	2·2	0·3	2·8	0·4
	CAT (µmol/min/mg prot)	24·1	3·1	33·5	7·6
	GR (mU/mg prot)	47·1	11·72	41·64	3·93
	GPx (mU/mg prot)	57·2	9·07	48·0	3·82
	GSH (nmol/mg prot)	33·4	7·4	36·7	3·7
	GSSG (nmol/mg prot)	0·5	0·1	0·6	0·1
	GSH/GSSG	71·0	18·1	64·5	8·6
Heart	SOD (U/g mg prot)	6·0	0·5	5·6	0·7
	CAT (µmol/min/mg prot)	15·1	0·6	13·4	1·1
	GR (mU/mg prot)	23·6	1·4	27·4	2·3
	GPx (mU/mg prot)	146·6	9·9	159·6	9·0
	GSH (nmol/mg prot)	21·5	2·8	18·1	2·1
	GSSG (nmol/mg prot)	6·4^**^	0·6	4·5	0·3
	GSH/GSSG	3·4	0·5	4·0	0·5
Brain	SOD (U/g mg prot)	8·0	0·2	7·4	1·5
	CAT (µmol/min/mg prot)	6·4^**^	0·9	2·2	0·7
	GR (mU/mg prot)	67·4	7·3	49·8	3·7
	GPx (mU/mg prot)	49·0^**^	5·3	30·9	1·5
	GSH (nmol/mg prot)	87·2^**^	16·5	24·2	7·3
	GSSG (nmol/mg prot)	5·2	0·4	3·0	1·0
	GSH/GSSG	16·8	3·4	8·0	3·5

1 SHROB, Spontaneously Hypertensive Obese; WKY, Wistar Kyoto; SOD, superoxide dismutase; CAT, catalase; GR, glutathione reductase; GPx, glutathione peroxidase; GSH, glutathione; GSSG, glutathione disulphide; Hb, haemoglobin.

Statistically significant differences between the samples are indicated by * (*P*<0.05), ** (*P*<0.01) and *** (*P*<0.001).

#### SOD and CAT activity

The activity of the endogenous antioxidant enzymes was measured in erythrocytes, liver, abdominal fat, kidney and heart. No significant differences in SOD activity between SHROB and WKY rats were observed in any of the samples analysed. As regards CAT activity, brain was the only organ where significant differences (*P*<0.01) were found between the SHROB and WKY rats.

#### Glutathione system

The status of the glutathione system was evaluated by quantifying the two forms of glutathione (GSH and GSSG) to calculate the GSH/GSSG ratio; and by measuring the activity of the associated enzymes (GR, which regenerates reduced glutathione; and GPx, which catalyses the oxidation of GSH to GSSG). Higher concentrations of GSH in liver (*P*<0.01), abdominal fat (*P*<0.05) and brain (*P*<0.01) were found in the SHROB rats than the WKY rats. GSH/GSSG was significantly (*P*<0.05) higher in the adipose tissue of the SHROB rats. GPx activity was also higher in the abdominal fat tissue (*P*<0.001) and brain (*P*<0.01) of the SHROB rats. In contrast, higher concentration of GSSG were found in the heart of the SHROB rats than in the WKY rats (*P*<0.01) and the SHROB rats showed lower GPx activity in erythrocytes and in liver (*P*<0.05) and lower GR activity in liver (*P*<0.05).

### Oxidative stress

Several biomarkers of OS were measured in blood, urine and different organs in the SHROB and WKY rats (**Table 3**). Plasma antioxidant capacity, as determined by ORAC assay, was significantly (*P*<0.05) higher in the SHROB than in the WKY rats. Lipid oxidation was evaluated by determining oxidized LDL in plasma, MDA in liver, kidney, abdominal fat, heart and brain, and 15-F_2t_-IsoP in urine. Oxidized LDL in plasma was 50% higher in the SHROB rats than in the WKY rats, although this increase did not reach significance, due to the high interindividual variability of this parameter. Similarly, isoprostane 15-F_2t_-IsoP in urine was non-significantly higher in the urine of SHROB rats. Creatinine was higher in SHROB rats than in WKY rats (68 mg/dL SEM 9 *vs* 58 mg/dL SEM 7); since 15-F_2t_-IsoP is expressed per mg of creatinine, the higher values of this isoprostane in the SHROB rats is not an artefact resulting from increased creatinine values. In contrast, MDA in abdominal fat was significantly lower (*P*<0.05) in SHROB rats than in WKY rats and overall protein oxidation-measured as total carbonyls-was lower in the SHROB than in the WKY rats in both the low and high ionic strength fractions of liver proteins (*P*<0.05) as well as in plasma (*P* = 0.0571).

**Table 3:** Oxidative stress biomarkers in SHROB and WKY rats (mean value and SEM)^1^.

Organ	Parameter	SHROB		WKY	
			SEM		SEM
Plasma	ORAC (µmol Trolox/mL)	44·8^*^	3·2	30·3	3·6
	LDL-ox (ng/mL)	154·6	21·4	109·0	10·2
	Overall protein oxidation (FTSC intensity)	78·9	12·0	136·0	2·4
Liver	MDA (nmol/mg prot)	9·9	0·8	8·1	1·0
	Overall low ionic strength protein oxidation (FTSC intensity)	51·3^*^	2·7	74·7	0·7
	Overall high ionic strength protein oxidation (FTSC intensity)	81·4*	6·9	295·7	23·6
Abdominal fat	MDA (nmol/mg prot)	5·6^*^	1·2	16·0	3·1
Kidney	MDA (nmol/mg prot)	0·7	0·1	0·8	0·1
Heart	MDA (nmol/mg prot)	4·8	0·8	4·3	0·9
Brain	MDA (nmol/mg prot)	28·7	10·0	18·2	1·2
Urine	15-F_2t_-IsoP (ng/mg creatinine)	2·9	0·2	1·9	0·1

1 SHROB, Spontaneously Hypertensive Obese; WKY, Wistar Kyoto; ORAC, oxygen radical absorbance capacity; MDA, malondialdehyde.

Statistically significant differences between the samples are indicated by * (*P*<0.05), ** (*P*<0.01) and *** (*P*<0.001).

## Discussion

SHROB rats represent a particularly interesting animal model for MetS, a situation prior to the onset of type-2 diabetes or CVD, in which the different metabolic alterations may be addressed without the use of drugs. The aim of the present study was to contribute to the description of this animal model, by examining parameters related to cardiovascular function, inflammation and oxidative stress not previously reported.

Total cholesterol, triglycerides, fasting glucose, insulin, glycated haemoglobin and blood pressure, have been examined before in SHROB rats [15], [17] and in the related SHRSP (stroke-prone spontaneously hypertensive rat) model, after supplementation with soybean oil [36]. Our results concord with previous reports except for glycaemia; some authors found hyperglycaemia [15], [37], [38] while others reported normoglycaemia, [17], [39], as we do. Anti-atherogenic ApoA1 and pro-atherogenic Apo B100 are now included in the lipid profile. Certain markers of endothelial dysfunction (ICAM-1 and VCAM-1), inflammation (C-reactive protein) and thrombotic activity (PAI-1) are also reported here for the first time.

The SHROB rats exhibited significant alterations in abdominal fat, plasma lipid profile (total cholesterol, LDL-cholesterol, HDL-cholesterol, triglycerides, apoA1, apoB100), insulin, blood pressure, ICAM-1, C-reactive protein and PAI-1 (**Table 1**). In many of these parameters (lipid profile, markers of endothelial dysfunction, inflammation and thrombotic activity), the values in the SHROB rats were at least 50% higher than in the WKY rats. Impairment of endothelial function in SHROB rats has previously been related to alterations in the nitric oxide pathway and the presence of visceral prostaglandins derived from perivisceral adipose tissue [36], [37] All these results are consistent with high circulating fat, visceral fat accumulation and the state of low-level inflammation that precedes insulin resistance [40]. Inflammation appears to be the link between obesity and the development of insulin resistance in MetS [41]. The mutation (fa^k^) in the leptin receptor of SHROB rats that impairs the ability of leptin to regulate fat intake would explain these effects with a direct relation to an excess of fat.

Since OS is a complex process, where there are different interactions between endogenous antioxidants systems, progress of oxidation in biomolecules and levels of circulating exogenous antioxidants [42], we decided to measure markers corresponding to each one of these components of OS. The picture that emerges from our results is of a marked difference between systemic OS (as measured via the endogenous antioxidant system in erythrocytes and oxidized LDL in plasma) and OS in organs (as measured via the endogenous antioxidant system in tissues and organs, and via final lipid and protein oxidation products in organs and in urine). Both SOD and GPx activities in erythrocytes were significantly reduced in the SHROB rats, and this attenuation of the endogenous defence system was concomitant with a 50% increase in LDL oxidation. This is consistent with the preceding observations related to CVD biomarkers, since OS, particularly low SOD activity, has been shown to be present in subjects with MetS who also show high C-reactive protein levels [4]. However, this systemic OS was not immediately translated to the different organs, since the levels of MDA in liver and kidney and of 15-F_2t_-IsoP in urine were not significantly modified in the SHROB rats. Indeed, compared to the WKY rats, the SHROB strain presented a significant increase in GSH in liver, abdominal fat and brain as well as a significant decrease in overall protein carbonylation and lipid oxidation in abdominal fat. We found that, in SHROB rats, OS may present later than other reported organ disturbances such as increased renal macrophage infiltration [43]. Also, it should be noted that even if total protein carbonylation in SHROB rats is shown to be lower than in WKY rats, we recently reported that key proteins involved in redox homeostasis, such as aldehyde dehydrogenase mitochondrial, protein disulphide-isomerase A3 or protein disulphide-isomerase, suffer higher oxidation in SHROB rats than in WKY rats [44]. Finally, the higher plasma antioxidant capacity in the SHROB rats, concordant with the lower protein carbonylation found in SHROB plasma may be the result of the activation of defence mechanisms that are not observed to have been overwhelmed in most organs. The observed differences between the different markers of OS, corresponding to different stages and elements of this process, emphasize the need to carry out this kind of integrated evaluations of OS in *in vivo* studies on metabolic syndrome.

The results presented here provide an updated picture of CVD-related markers in SHROB rats. In blood, the animals show elevated levels of markers related to inflammation, endothelial dysfunction, thrombotic activity, insulin resistance and oxidative stress (C-reactive protein, total cholesterol, LDL-cholesterol, triglycerides, apoA1, apoB100, abdominal fat, blood pressure, ICAM-1 and PAI-1, insulin and oxidized LDL). In other organs (abdominal fat, liver and brain) the glutathione system is activated and oxidative stress remains low, according to the observed concentrations of final lipid and protein oxidation products. All these observations are consistent with a pre-diabetic obese status of SHROB rats with a high risk of developing CVD that may be used to evaluate the effects of nutritional interventions on the metabolic alterations present in MetS.

## Supporting Information

Figure S1 **Standard curve used to quantify GSH (glutathione).**
(DOC)

Figure S2 **Standard curve used to quantify GSSG (glutathione disulphide).**
(DOC)

Figure S3 **Representative chromatogram of endogenous 15-F_2t_-IsoP in rat urine.** A) The *m/z* 573 ion current chromatogram represents the [^2^H_4_]-15-F_2t_-IsoP internal standard; the signal used to quantify 15-F_2t_-IsoP. B) The *m/z* 569 ion current chromatogram represents endogenous 15-F_2t_-IsoP. The injection volume was 1 µL.
(DOC)

Figure S4 **Evolution of the body weight of SHROB and WKY rats from week 13 to week 27 of life.**
(DOC)

Table S1 **Fatty acid composition of the soybean oil provided weekly.** Values are given as mean ± SD.
(DOC)
