# Supplementary material for: Cardiovascular Disease-Related Parameters and Oxidative Stress in SHROB Rats, a Model for Metabolic Syndrome
Source: PLoS One. 2014 Aug 12;9(8):e104637. doi: 10.1371/journal.pone.0104637 (PMC4130542; doi:10.1371/journal.pone.0104637)
Supplement: Figure S1 — Standard curve used to quantify GSH (glutathione). (DOC) [file pone.0104637.s001.doc]

**Figure S1.** Standard curve used to quantify GSH (glutathione)
