# Supplementary material for: Cardiovascular Disease-Related Parameters and Oxidative Stress in SHROB Rats, a Model for Metabolic Syndrome
Source: PLoS One. 2014 Aug 12;9(8):e104637. doi: 10.1371/journal.pone.0104637 (PMC4130542; doi:10.1371/journal.pone.0104637)
Supplement: Figure S3 — Representative chromatogram of endogenous 15-F2t-IsoP in rat urine. A) The m/z 573 ion current chromatogram represents the [2H4]-15-F2t-IsoP internal standard; the signal used to quantify 15-F2t-IsoP. B) The m/z 569 ion current chromatogram represents endogenous 15-F2t-IsoP. The injection volume was 1 µL. (DOC) [file pone.0104637.s003.doc]

**Figure S3**. Representative chromatogram of endogenous 15-F_2t_-IsoP in rat urine. A) The *m/z* 573 ion current chromatogram represents the [^2^H_4_]-15-F_2t_-IsoP internal standard; the signal used to quantify 15-F_2t_-IsoP. B) The *m/z* 569 ion current chromatogram represents endogenous 15-F_2t_-IsoP. The injection volume was 1 µL.
