# Supplementary figures and images for: Cardiovascular Disease-Related Parameters and Oxidative Stress in SHROB Rats, a Model for Metabolic Syndrome
Source: PLoS One. 2014 Aug 12;9(8):e104637. doi: 10.1371/journal.pone.0104637 (PMC4130542; doi:10.1371/journal.pone.0104637)

**Figure S4.** Evolution of the body weight of SHROB and WKY rats from week 13 to week 27 of life


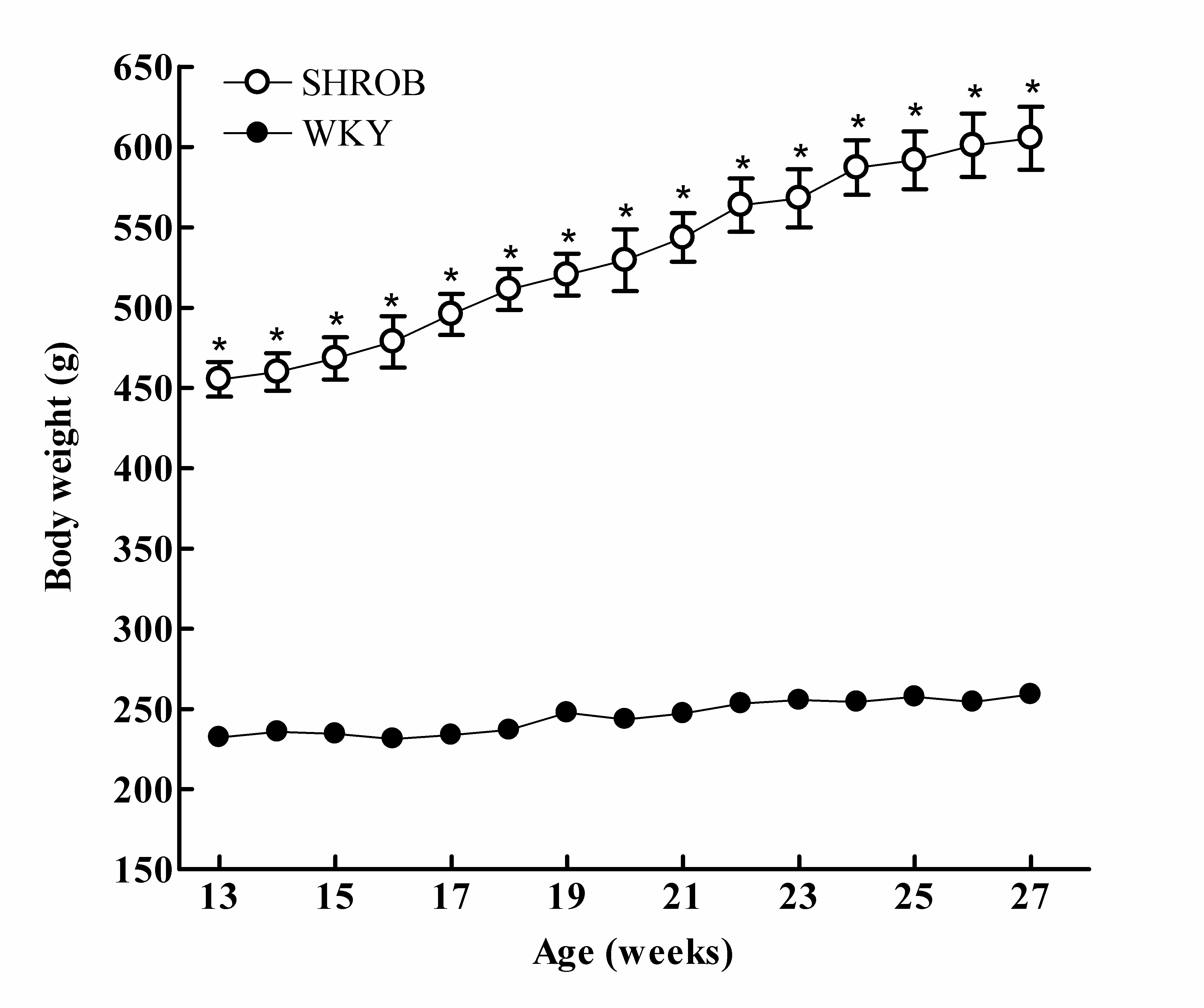

Supplement: Figure S4 — Evolution of the body weight of SHROB and WKY rats from week 13 to week 27 of life. (DOC) [file pone.0104637.s004.doc]
