# Supplementary material for: Cardiovascular Disease-Related Parameters and Oxidative Stress in SHROB Rats, a Model for Metabolic Syndrome
Source: PLoS One. 2014 Aug 12;9(8):e104637. doi: 10.1371/journal.pone.0104637 (PMC4130542; doi:10.1371/journal.pone.0104637)
Supplement: Table S1 — Fatty acid composition of the soybean oil provided weekly. Values are given as mean ± SD. (DOC) [file pone.0104637.s005.doc]

**Table S1**. Fatty acid composition of the soybean oil provided weekly. Values are given as mean ± SD.

|  | Content (mol %) |
| --- | --- |
| 14:0 | 0.96 ± 0.02 |
| 15:0 | 0.15± 0.01 |
| 16:0 | 17.78 ± 0.10 |
| 16:1ω7 | 0.90 ± 0.03 |
| 17:0 | 0.21 ± 0.01 |
| 18:0 | 2.07 ±0.01 |
| 18:1ω9 | 18.75 ± 0.03 |
| 18:1ω7 | 1.52 ± 0.02 |
| 18:2ω6 | 47.55 ± 0.01 |
| 18:3ω3 | 4.00 ± 0.04 |
| 20:1ω9 | 1.43 ± 0.09 |
| 18:4ω3 | 0.15 ± 0.004 |
| 20:2ω6 | 0.20 ± 0.05 |
| 20:4ω6 | 0.40 ± 0.02 |
| 22:1ω11 | 1.08 ± 0.005 |
| 22:1ω9 | 0.25 ± 0.02 |
| 20:4ω3 | 0.20 ± 0.03 |
| 20:5ω3 | 0.70 ± 0.02 |
| 24:1ω9 | 0.28 ± 0.05 |
| 22:5ω3 | 0.26 ± 0.01 |
| 22:6ω3 | 1.15 ± 0.03 |
| **ω3** | 6.47 ± 0.14 |
| **SFA** | 21.17 ± 0.10 |
| **MUFA** | 24.21 ± 0.11 |
| **PUFA** | 54.62 ± 0.03 |
| **EPA+DHA** | 1.85 ± 0.06 |

ω3, omega-3; SFA, saturated fatty acids; MUFA, monounsaturated fatty acids; PUFA, polyunsaturated acids.
